# Assesment of pregnancy outcomes among twin pregnancies with single fetal demise regarding chorionicity and fetal death time

**DOI:** 10.4274/jtgga.galenos.2018.2018.0053

**Published:** 2019-08-28

**Authors:** Sevcan Arzu Arınkan, Resul Arısoy, Murat Api

**Affiliations:** 1 Department of Obstetrics and Gynecology, İstanbul Zeynep Kamil Maternity and Pediatric Training and Research Hospital, İstanbul, Turkey

**Keywords:** Single twin demise, intrauterine death, twin pregnancy, perinatal outcomes, pregnancy outcomes

## Abstract

**Objective:**

The objective of this study was to assess maternal and perinatal outcomes of twin pregnancies with single fetal demise in terms of chorionicity and fetal death time.

**Material and Methods:**

All deliveries between January 2008 and July 2015 were reviewed retrospectively and 85 twin pregnancies with single fetal demise were included. These cases were grouped according to chorionicity and fetal death time.

**Results:**

The incidence of single fetal demise was 4.7%. The mean delivery week was later in the dichorionic group (34.16±4.65) than in the monochorionic group (31.1±3.83). The ratios of deliveries before the 34^th^ gestational week were 71.4% in monochorionics and 35% in dichorionics. Monochorionics had a 13 times greater risk for having delivery before the 37^th^ gestational week and a 4 times greater risk for having delivery before the 34^th^ gestational week compared with dichorionics. Furthermore, monochorionics had a 7 times greater risk for having abruptio placenta compared with dichorionics. The newborn intensive care unit admission ratios were 61.3% in dichorionics and 85.7% in monochorionics. Also, monochorionics had a 3.7 times greater risk for admission to newborn intensive care unit compared with dichorionics.

**Conclusion:**

We recommend follow-up of twin pregnancies with single fetal demise in terms of premature birth, regardless of chorionicity. Also, close monitoring is recommended for monochorionic twin pregnancies with single fetal demise in terms of premature birth before 34 weeks of gestation, abruptio placenta, the need for neonatal intensive care, and respiratory distress syndrome.

## Introduction

In monozygotic twins compared with dizygotic twins pregnancy, the relative risks of exitus of two fetuses, single fetal demise, and neonatal exitus of a living fetus were reported as 20, 1.63, and 2.26, respectively (1). The incidence of single fetal demise after the 20^th^ week among all twin pregnancies ranges from 2.6% to 6.2% (2). Chorionicity is an important factor in the ratio of intrauterine loss; the risk of fetal demise is greater in monochorionic twin pregnancies compared with dichorionic twin pregnancies (3). One of the main reasons for this situation is anastomoses of placental circulation and twin-to-twin transfusion syndrome risk (4). Intrauterine death of one fetus significantly increases the risk of mortality and morbidity of the living fetus (4). The management after single fetal demise is considered according to chorionicity and gestational age. The decision for delivery should be given by considering prematurity-related complications or morbidity and mortality that may be seen in the living fetus. If there are no other obstetric causes, delivery of dichorionic twin pregnancies with single fetus demise is not recommended before the 38^th^ week (4). However, regular monitoring of living twin growth and follow-up in terms of hypertension, preeclampsia, and coagulopathy is recommended (5,6). In monochorionic twin pregnancies with single fetal demise, premature birth, intrauterin exitus or ischemic brain injury risks are present for the living twin (5,6). Ischaemic brain damage is considered to occur during or immediately after single fetal demise (6). For this reason, follow-up is recommended to avoid prematurity-related complications before 34 weeks in monochorionic pregnancies after single fetal demise (5). Barigye et al. (6)reported that the risk of fetal loss in the third trimester was also high in uncomplicated monochorionic pregnancies. They calculated that 23 cases in the 32^nd^ week and 30 cases in 34^th^ week should be delivered to save one fetus (7).

The objective of this study was to assess maternal and perinatal outcomes of twin pregnancies with single fetal demise in terms of chorionicity and fetal death time.

## Material and Methods

All the deliveries between January 2008 and July 2015 were reviewed retrospectively and 85 twin pregnancies with single fetal demise were included in the study. Monoamniotic pregnancies, pregnancies with both fetal demises, singleton gestations, higher-order multiple gestations, pregnancies discontinued antenatal surveillance, and cases which chorinocity that was not exactly determined were excluded. Only pregnancies with complete outcome information were included. These cases were grouped according to chorionicity and fetal death time (0-13, 14-28, 29-34 gestational weeks). Chorionicity was determined using the earliest available ultrasound or confirmed by pathology. Gestational age was determined by the first day of a woman’s last menstrual period and with the earliest ultrasound. Data were controlled for gestational age at delivery. Antenatal steroids and tocolitics were administered between 24 and 34 weeks if delivery was expected within 7 days. The criteria for deliveries were spontaneous preterm delivery, preeclampsia, deterioration of Doppler, and non-reassuring cardiotocography. Dichorionic diamniotic pregnancies were compared with monochorionic diamniotics regarding to preeclampsia, gestational diabetes (GDM), abruptio placenta, preterm delivery (34 and 37 gestational weeks), premature rupture of membranes (PROM), intrauterine growth retardation (IUGR). IUGR was diagnosed when esimated fetal weight was below the 10th percentile for gestational age. A 50 g oral glucose test was performed to all patients. If the screening test was positive (>140 mg/dL), a 3 hours’ glucose tolerance test was performed. GDM diagnosis was confirmed with any two abnormal values (≥95-180-155-140 mg/dL). Also, intensive care unit admission, intracranial hemorrhage, phototherapy, polycythemia, respiratory distress syndrome (RDS), sepsis, patent ductus arteriosus (PDA), bronchopulmonary dysplasia (BPD), and twin-to-twin syndrome (TTTS) were also studied. Capillary hematocrit was sampled at 12 hours after birth. Venous hematocrit was obtained from those with hematocrits more than 70%. Venous hematocrits more than 65% were accepted as polycythemic.

The study was approved by the Ethics and Clinical Investigation Committee. The Statistical Package for the Social Sciences (SPSS; Version 20.0, Chicago, IL, USA) was used for statistical analyses. Descriptive statistics are presented as mean ± standard deviation for normally distributed data, and as numbers and percentages for categorical data. The relationship between the categorical variables was examined using the chi-square test and Fisher’s exact test. The results were evaluated with a confidence interval of 95%, and p<0.05 / p<0.01 was considered statistically significant. The Kolmogorov-Smirnov test was used for the assessment of the normality of data. The Mann-Whitney U test was used for data that were not normally distributed.

## Results

Between January 2008 and July 2015, 1808 of a total of 77,204 deliveries were twins (2.34%); 85 twin pregnancies with single fetal demise were included in the study. Single fetal demise was seen in about 4.7% of pregnancies. The average age of patients participating in the study was 29±6 years. Seventy-four percent of cases (n=64) were diamniotic dichorionic twin pregnancies, and 26% (n=21) were diamniotic monochorionic twin pregnancies. In addition, 19% (n=16), 40% (n=34), and 41.2% (n=35) of fetal demise occured in the first, second, and third trimesters, respectively. The average gestational week for delivery was 34 weeks and birth weight was 2099±795 g.

The average gestational age for delivery in dichorionic twin pregnancies (34.3±4.6 weeks), which was higher than the average gestational age in monochorionics (32±4 weeks, p=0.009). The average birthweight was 2.222±835 g in dichorionic twin pregnancies and 1.836±627 g in monochorionic twin pregnancies (p=0.052).

Preeclampsia was observed in 27.4% (n=17) of the dichorionic twin pregnancy group and 28.6% (n=6) monochorionic twin pregnancies (p=0.919). The distribution of patients with preeclampsia in the dichorionic group was 35.3%, 41.2%, and 23.5% in that single fetal demise was seen in the first, second, and third trimesters, respectively. Monochorionic and dichorionic groups were compared according to preeclampsia and fetal death time and there was no statistically significant difference (p>0.05) (Table 1).

The incidence of abruptio placenta was higher in monochorionic twin pregnancies (19%) compared with dichorionic twin pregnancies (3.2%) and the incidence of abruptio placenta was 7 times higher in monochorionic twin pregnancies compared with dichorionic twin pregnancies [odds ratio (OR): 7.05; p=0.033]. The distribution of pregnancy complications according to chorionicity is shown in Table 1.

Premature rupture of membranes was seen in 16.1% (n=10) of cases in the dichorionic group and in 9.5% (n=2) of cases in the monochorionic group. The incidence of IUGR was 11.3% (n=7) in the dichorionic group and 14.3% (n=3) in the monochorionic group. There was no statistically significant difference between monochorionic and dichorionic twin pregnancies in terms of the incidence of premature rupture of membranes, IUGR, and oligohydramnios (p>0.05). There was no statistically significant difference between the monochorionic and dichorionic groups according to the fetal death time in terms of incidence of IUGR and PROM (p>0.05). The distribution of pregnancy complications according to chorionicity and fetal death time are shown in Table 2.

The frequency of deliveries before the 37^th^ gestational week after the death of one twin was found to be 13 times higher in monochorionic twin pregnancies than in dichorionics (OR: 13.33, p=0.002). The frequency of delivery before the 34th gestational week after the death of one twin was found to be 4 times higher in monochorionic twin pregnancies than in dichorionics (OR: 4.64, p=0.005). In the dichorionic group, there was a statistically significant difference in terms of time of fetal demise (34^th^ week and 37^th^ week) (p=0.012, p=0.002). In the dichorionic group, the rate of giving birth before the 37^th^ gestational week was found to be higher in those with single fetal demise in the second trimester (81%) compared with the third trimester (59%) and first trimester (38%) (p=0.041) (Table 2).

The ratio of newborns whose 1-minute APGAR score was less than 7 was found higher in the monochorionic group (74%) compared with the dichorionic group (51%). Similarly, the ratio of patients whose 5-minute APGAR score was less than 7 was found higher in the monochorionic group (47.1%) compared with the dichorionic group (13.7%). Although the 5-minute APGAR score showed statistically significant differences according to chorionicity, there was no statistically significant difference for the 1-minute APGAR score (p=0.007 and p=0.086).

The need for neonatal intensive care was 61.3% in the dichorionic group and this ratio was 86% in the monochorionic group. The incidence of RDS was 25% and 47% in the dichorionic and monochorionic groups, respectively (p=0.095) (Table 3). The need for neonatal intensive care was 3.7 times greater in monochorionic pregnancies compared with dichorionic pregnancies (OR: 3.78, p=0.039).

The incidence of sepsis was 17.6% (n=6) in the dichorionic group, and 35.7% (n=5) in the monochorionic group. However, there was no statistically significant difference between the groups according to chorionicity in terms of sepsis, hypoglycemia development, and phototherapy (p>0.05). The comparison of neonatal complications by chorionicities is shown in Table 3.

In the dichorionic group, the distribution of patients with neonatal intensive care needs was as follows: 52.6%, 34.2%, and 13.2% in the group with single fetal demise in the second, third, and first trimesters, respectively. In the monochorionic group, 55.6% of patients requiring neonatal intensive care were in the group with single fetal demise in the third trimester. There were statistically significant differences between the dichorionic and monochorionic groups accrding to time of single fetal demise in terms of frequency of neonatal intensive care need (p=0.006 and p=0.043). Neonatal outcomes and the distributions of pregnancies by time of fetal demise are shown in Table 4.

In the dichorionic group, 91% of patients with RDS were in the group with single fetal demise in the second trimester. In the monochorionic group, the distribution of patients with RDS was 75% and 25% in the group with single fetal demise in the second and third trimesters, respectively. There were statistically significant differences in terms of RDS according to fetal death time in the dichorionic and monochorionic groups (p=0.001 and p=0.008) (Table 4).

In the dichorionic group, the distribution of 21 patients who underwent phototherapy was as follows: 62% and 24% were in the group with single fetal demise in the second and third trimesters, respectively. This difference was statistically significant (p=0.005) (Table 4).

The mean hemoglobin level in fetuses after delivery was 49.83 g/dL. Polycythemia was identified in 6 patients. There was no statistically significant difference in terms of incidence of polycythemia by chorionicity. Three and one of four patients in the dichorionic group were in the group of single fetal demise in the first and third trimesters, respectively. This difference was found statistically significant (p=0.005).

BPD was monitored in a total of 4 newborns, 3 and 1 of the patients were dichorionic and monochorionic twin pregnancies, respectively. PDA was seen in 6 neonates (5 dichorionic, 1 monochorionic). Intracranial hemorrhage was detected in 5 patients including 3 and 2 patients in dichorionic and monochorionic groups, respectively. There was no significant difference between the groups in terms of intracranial hemorrhage, BPD, and PDA regarding chorionicity and fetal demise time (p>0.05). TTTS was identified in a total of 7 patients, 6 of them stage 1, and 1 one was stage 3. Laser ablation was performed to one patient.

Consumption coagulopathy was observed in no cases.

## Discussion

The risk of morbidity and mortality in living fetuses can be explained by hemodynamic temporary fluctuations between twins and the theories of embolism and coagulopathy between the chorions (8). It is proposed that this coagulopathy in the living twin can lead to infarctions, and cystic changes in renal, pulmonary, hepatic, splenic and neurologic systems (7). Single fetal demise in the second and third trimester was seen in approximately 0.5-6% of twin pregnancies (8). Consistent with the literature, in our study, single fetal demise was seen in approximately 4.9% of twin pregnancies, single fetal demise after the first trimester was seen in 4.01%. Although there are insufficient data about the adverse effects for the living twin after single fetal demise in the first trimester, this subject is still controversial. Sun et al. (9) detected lower birthweight in pregnancies with vanishing twin syndrome compared with single pregnancies in their. In addition, brain abnormalities have been shown in the living twin in monochorionic twins with single fetal demise in the first trimester (10,11).

Ong et al. (2) detected preterm birth before 34 weeks as 68% in monochorionic twins with single fetal demise and 57% in dichorionics in their systematic review. In addition, it has been reported that premature birth before 34 weeks was more common in monochorionic twin pregnancies with single fetal demise, but this difference was not statistically significant (2). Aslan et al. (12) reported without distinction of chorionicity the premature birth rate as 81.3% and 41.6% for the 37^th^ and 32^nd^ gestational weeks, respectively.

Frequency of preterm delivery as 46% and 43% among monochorionic and dichorionic twin pregnancies, respectively. They concluded that the difference between the groups was not statistically significant, but noted a negative correlation between the mean gestational week of fetal death and the mean gestational week at delivery. Furthermore, no significant correlation was found between the mean gestational week of fetal death and mean fibrinogen levels.

Giwnewer et al. (13) reported the rate of premature birth before the 37^th^ and 34^th^ weeks in diamniotic pregnancies with single fetal demise as 73.3% and 38.8%, respectively. Unlike in our study, we found a higher birth rate before 34 weeks in the monochorionic group. In addition, we detected that the frequency of deliveries before 37 weeks was 11 times greater in monochorionic twin pregnancies after single fetal demise than in dichorionics, and the frequency of delivery before 34 weeks was 4 times greater. In addition, we found the frequency of deliveries before 37 weeks of dichorionic twin pregnancies with single fetal demise in the second trimester (81%) higher than the third and first trimesters. Different from the literature, we found differences in the frequency of deliveries before both the 37^th^ and 34^th^ weeks according to chorionicity. Also, in addition to the information in the literature, we found the frequency of deliveries before 37 weeks in dichorionic pregnancies with single fetal demise in the second trimester was higher than pregnancies with single fetal demise in the third (28-34^th^ gestational weeks) and first trimesters.

Giwnewer et al. (13) detected premature rupture of membranes as 6% in diamniotic twin pregnancies with single fetal demise in their study performed without discriminating chorionicity. Fichera et al. (14) reported PPROM in one patient in their dichorionic group at the 33^rd^ gestational week. We found no statistically significant difference in terms of incidence of PROM by chorionicity.

Fichera et al. (14) detected preeclampsia in their study consisting of 23 cases of single fetal demise in the second and third trimesters in one (7.7%) patient in the monochorionics group, which consisted of 13 patients, and two (20%) in the dichorionic group of 10 patients. Aslan et al. (12) found preeclampsia in 3 (9.4%) of 32 cases in their study, which was performed without chorionicity discrimination. In the study conducted by Giwnewer et al. (13), mild and severe preeclampsia rates were detected as 8.6% and 5.2%, respectively, in diamniotic pregnancies with single fetal demise. In our study, the incidence of preeclampsia was higher in contrast to the literature. We observed no differences in terms of incidence of preeclampsia by chorionicity. Deveer et al. (15) detected preeclampsia in 2 patients, one of them was in the first trimester group and the other was in the group of first trimester and after the first trimester. In our study, we found no statistically significant difference in terms of incidence preeclampsia and PROM by fetal death time in monochorionic and dichorionic groups.

In the study conducted by Giwnewer et al. (13) abruptio placenta was detected as 0.09% in diamniotic pregnancies with single fetal demise, and 1.9% in the control group, which comprised diamniotic twin pregnancies. This difference was not statistically significant. In our study different from Giwnewer et al. (13), abruptio placenta rate was detected as 3.6% (n=2) in dichorionic twin pregnancies and 20% (n=4) in the monochorionic group. In addition, the incidence of abruptio placenta was more than 6 times higher in monochorionic twin pregnancies than in dichorionic twin pregnancies.

In the study conducted by Giwnewer et al. (13) intrauterine growth retardation was detected in 3.4% of fetuses in diamniotic pregnancies with single fetal demise and postpartum death was detected in 9.5% of diamniotic pregnancies with single fetal demise. Chelli et al. (16) assessed 33 cases with single fetal demise after the 26^th^ gestational week, postpartum death was detected in 6 patients.

Giwnewer et al. (13) detected the average birthweight of diamniotic pregnancies with single fetal demise as 1953 g and the proportion of those with low birthweight (<2500 g) as 71.6%. In addition, the proportion of patients with 1-minute APGAR score less than 7 and 5-minute APGAR score less than 7 was found as 30% and 6.9%, respectively. In our study, the proportion of patients with 1-minute APGAR scores less than 7 in the dichorionic group (47.9%) was found to be lower than the monochorionic group (72.2%). In addition, the proportion of patients with 5-minute APGAR scores less than 7 in dichorionic group (15.2%) was found to be lower than the monochorionic group (43.8%). In our study, there was a statistically significantly difference in terms of 1-minute APGAR scores by chorionicity, but there was no statistically significantly difference in terms of 5-minute APGAR scores.

Deveer et al. (15) reported the need for neonatal intensive care in 5 of 38 patients in their study. All of these patients were in the group with single fetal demise after the first trimester. In our study, the risk of need for neonatal intensive care was 3.4 times higher in monochorionic pregnancies than in dichorionic pregnancies. Similar to the study conducted by Deveer et al. (15), we also found that 48,6%, 37.1%, and 14.3% of cases requiring neonatal intensive care in the dichorionic group were in the groups with single fetal demise in the second, third, and first trimesters, respectively. There was a statistically significant difference between the dichorionic and monochorionic groups in terms of the frequency of need for neonatal intensive care by fetal death time.

Reported that severe cerebral injury was diagnosed in 13 (26%) of 50 co-twins. They concluded that cerebral injury was due to hypoxic-ischemic injury resulting in cystic PVL, middle cerebral artery infarction or injury to basal ganglia, thalamus and/or cortex.

One of the most important outcome of fetuses in twin pregnancy with one IUFD is the neurologic condition of the surviving fetus, especially in monochorionic twins. Data about the MCA Doppler in the surviving fetus after one IUFD were not collected by chart reviews because only pregnancies with complete outcome information were included. This manuscript does not discuss these problems.

In our study, delivery before 37 and 34 weeks was found to be more frequent in monochorionic twin pregnancies with single fetal demise than in dichorionics. Furthermore, abruptio placenta, need for neonatal intensive care, and incidence of RDS were found to be higher in monochorionic twin pregnancies with single fetal demise than in dichorionic twin pregnancies with single fetal demise. We found the average gestational age at delivery as 34 weeks. We recommend follow-up of twin pregnancies with single fetal demise in terms of premature birth, regardless of chorionicity. Also, close monitoring is recommended for monochorionic twin pregnancies with single fetal demise in terms of premature birth before 34 weeks of gestation, abruptio placenta, the need for neonatal intensive care, and RDS.

## Figures and Tables

**Table 1:**
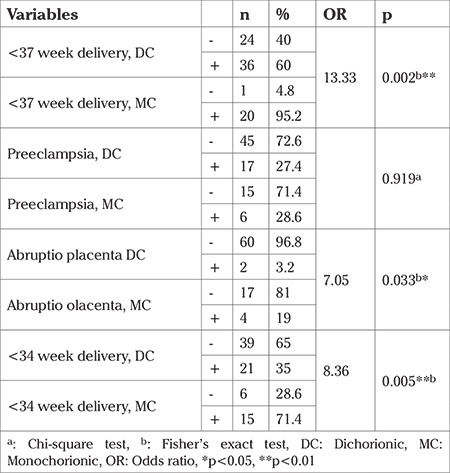
Obstetric outcomes regarding chorionicity

**Table 2:**
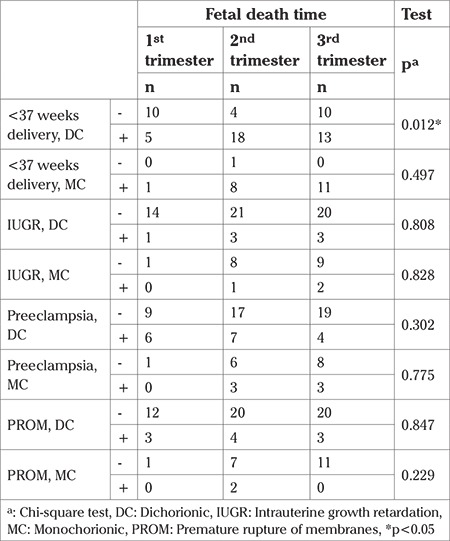
Obstetric outcomes regarding chorionicity and fetal death time

**Table 3:**
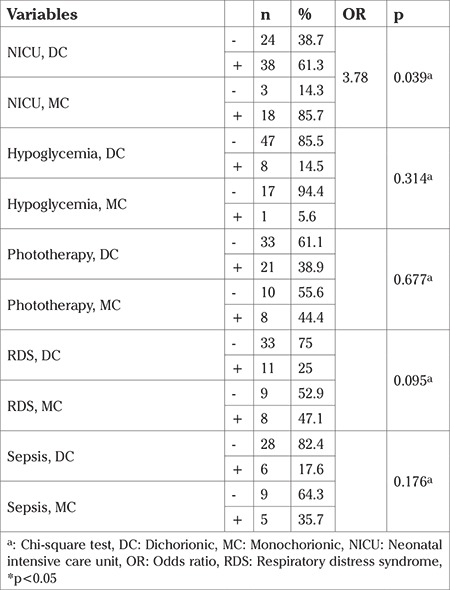
Fetal outcomes regarding chorionicity

**Table 4:**
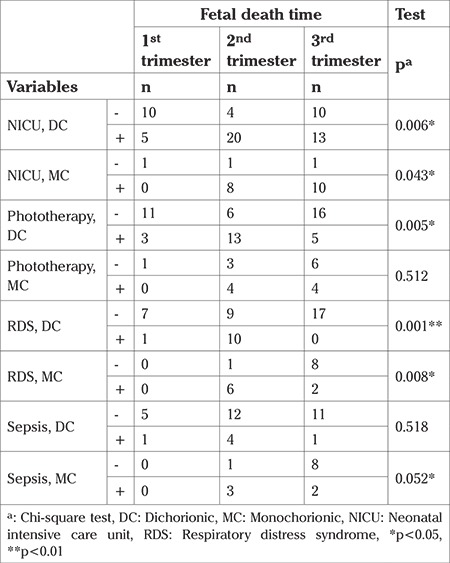
Fetal outcomes regarding chorionicity and fetal death time
